# Safety culture in intensive care in france: is there a link with morbi-mortality conferences?

**DOI:** 10.1186/2197-425X-3-S1-A427

**Published:** 2015-10-01

**Authors:** C Bretonnière, C Guitton

**Affiliations:** CHU Nantes, Nantes, France

## Introduction

Improving the safety of care is a challenge for the health systems. Development of a safety culture (SC) is one of the objectives. SC can be defined as a coherent and integrated set of individual and organizational behavior, based on shared values and beliefs, which continually seeks to reduce damage to patients, which may be related to health care. By "coherent and integrated set of behaviors", it refers to ways of acting, common practices, but also ways of feeling and thinking shared by professionals about security of care.

Morbidity-mortality conferences (MMC) are one tool that could help for improving SC.

The main objective of the national multicenter REA-C-SUR project is to measure the SC in ICUs in France. The secondary objective is to establish a link between SC and the characteristics of MMR.

This project, was initiated by the national group ‘RMM, Qualité et Sécurité des Soins en Réanimation’. It is funded by a national grant AO PREPS 2012.

## Material and Methods

SC was measured among professionals (doctors, managers, nurses...) through the questionnaire (Hospital Survey on Patient Safety Culture, HSOPS) developed by AHRQ in the United States and validated in French. This questionnaire, through 40 items, explores ten dimensions of SC (Table 1). A dimension is developed if the score is above 75% and "to be improved" if the score is less than 50%.

**Table 1:** HPOSP (French version): Dimensions.

1	Overall Perceptions of Patient Safety
2	Frequency of Events Reported
3	Supervisor/Manager Expectations & Actions Promoting Patient Safety
4	Organizational Learning—Continuous Improvement
5	Teamwork Within Units
6	Communication Openness
7	Non-punitive Response to Errors
8	Staffing
9	Management Support for Patient Safety
10	Teamwork Across Units

For each unit, MMCs’ organizational characteristics were collected.

## Results

From September 2013 to September 2014, 64 units (adults or pediatric) were included from French hospitals as follow: 40 teaching, 23 non-teaching, 1 private sector. A total of 36 149 patients, in 2012, were admitted.

**Figure 1:**
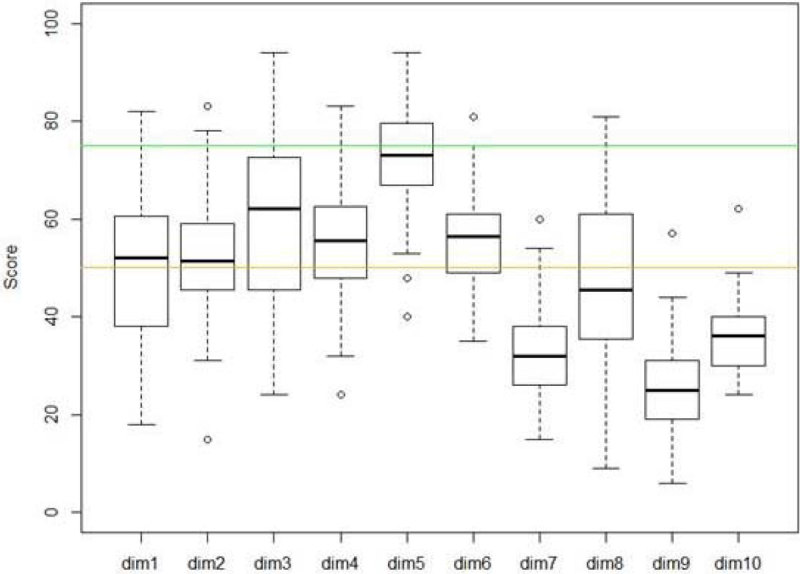
**Box-plots featuring scores for the 10 di.**

Participation rate was excellent: 3692 questionnaires filled out of 4811 (77%). SC overall is really undeveloped. The scores for perception by professionals in the management support for security (dim 9) or teamwork between departments (dim 10) are extremely bad: less than 50% for almost all units. The dimension "non-punitive response" is also very developed (Figure 1).

There is a statistical link between MMR and SC. The more MMR are structured, the better the scores for the overall perception of safety and support of management. There is also a link between nurses' involvement and reporting of adverse events, learning organization and human resources. Finally, the presence at MMCs, of an external participant, seems to improve communication openness.

## Conclusions

This prospective, multicenter study is the first in ICUs, in Europe. It might help in improving SC that is to date really low.

